# Supplementary material for: Empirical methods for controlling false positives and estimating confidence in ChIP-Seq peaks
Source: BMC Bioinformatics. 2008 Dec 5;9:523. doi: 10.1186/1471-2105-9-523 (PMC2628906; doi:10.1186/1471-2105-9-523)
Supplement: Additional File 1 — A variety of html documents from the USeq web site detailing the available applications, their best usage, output file type descriptions, command line menus, etc. [file 1471-2105-9-523-S1.zip › USeqUserGuides/JavaDoc.zip!/JavaDoc/trans/main/package-summary.html]

trans.main


|  |  |  |
| --- | --- | --- |
| **Overview** \| **Package** \| Class \| **Use** \| **Tree** \| **Deprecated** \| **Index** \| **Help** | |  |
| **PREV PACKAGE**   **NEXT PACKAGE** | **FRAMES**    **NO FRAMES**     **All Classes** |  |


---

## Package trans.main

Principle classes and scripts used in TiMAT2 analysis.

**See:**
  
          **Description**

| **Class Summary** | |
| --- | --- |
| **BindingPeak** | Holds information about a binding peak within an interval. |
| **BindingRegionGraphPrinter** | For printing chromosome specific .sgr files from IntervalPloter .txt picks files. |
| **ChromSet** |  |
| **ExportIntervalData** |  |
| **FDRVsQValues** |  |
| **FDRWindowConverter** | Associates an empirical FDR estimate with each window provided a mock IP was performed. |
| **FindSubBindingRegions** | Finds the best sub window and binding peaks (using the `PeakPicker`) within each interval. |
| **IntensityPrinter** | Prints out processed '.celp' files as '.sgr' files for import into Affy's IGB. |
| **Interval** | Container for holding information about a potential enriched region: windows, oligos, sub windows, binding peaks.... |
| **IntervalComparator** | Class to help sort an array of Interval[] by chromosome, start position, length shortest first. |
| **IntervalFilter** | Sorts an Interval[] into two groups, those that pass or fail a variety of filters. |
| **IntervalGFFPrinter** | Prints intervals as a GFF3 File. |
| **IntervalGraphPrinter** | Prints an array of Interval as an '.sgr' file for import into IGB. |
| **IntervalMaker** | Collapses an array of Window into and array of Interval based on scores. |
| **IntervalReportPrinter** | Prints all things contained within an interval to a spreadsheet or a report card. |
| **LoadChipSetIntervalOligoInfo** | Loads arrays of Interval[] with oligo and sequence information needed for other applications. |
| **LoadIntervalOligoInfo** | Loads arrays of Interval[] with oligo and sequence information needed for other applications. |
| **MakeAllWindows** | Makes all windows given oligo positions. |
| **MergeWindowArrays** |  |
| **MultiSGRPrinter** | Prints a multi sgr file (chr position val1, val2, val3, ....) |
| **MultiWindowIntervalMaker** | Collapses arrays of Windows into an array of Interval based on scores and minimal number of windows that must pass. |
| **Oligo** | Container to hold information about a particular oligo. |
| **OligoIntensityPrinter** | Prints out the oligo intensity values, split by chromosome, as '.sgr' files for import into Affy's IGB. |
| **OverlapCounter** | Provides an intersection analysis on multiple Interval[]s. |
| **PeakPicker** | Finds peaks in `Interval`s, both flat topped or peaks with sloping sides. |
| **PosRegion** | For holding information about a genomic interval, what genes are up, down, overlap, or contain this genomic inteval. |
| **RandomizeLabels** |  |
| **ReplaceWindowScores** |  |
| **SamChromosomeData** |  |
| **ScanChip** | Scores windows of intensity values using several tests. |
| **ScanChromosomes** | Scores windows of intensity values using several tests. |
| **ScanChromosomesCNV** | Scores windows of intensity values using several tests. |
| **ScanGenes** | Given a list of genes and their exons extracts the associated intensities. |
| **ScoredGene** |  |
| **ScoreIntervals** | Scores Intervals for the presence of transcription factor binding sites. |
| **SetNumberIntervalMaker** | Determines the threshold(s) needed to generate a set number of intervals, multiple score indexes accepted. |
| **SplitWindowArraysByChromosome** |  |
| **SubWindow** | Class to hold information about a subWindow. |
| **T2** | Runs entire TiMAT2 suite based on a parameter file. |
| **T2Parameter** | Container with info related to runing multiple T2 programs. |
| **TestScanChip** | Scores windows of intensity values using several tests. |
| **WilcoxonRankSumTest** | Implementation of a Wilcoxon Rank Sum Test. |
| **WilcoxonSample** | Used by the `WilcoxonRankSumTest`. |
| **WilcoxonSamplePair** | Used by the `WilcoxonSignedRankTest`. |
| **WilcoxonSignedRankTest** | Implementation of a Wilcoxon Signed Rank Test. |
| **Window** | Class to hold information about a window, a defined portion of the genome typically < 1kb. |
| **WindowBlockMaker** | Converts a Window[] into a heat map specific sgr file for import into IGB. |
| **WindowBlockMakerTwoColor** | Converts a Window[] into a heat map specific bar file for import into IGB. |
| **WindowComparator** | Class to help sort an array of Window[] by chromosome, start position, length shortest first. |

## Package trans.main Description

Principle classes and scripts used in TiMAT2 analysis.

---


|  |  |  |
| --- | --- | --- |
| **Overview** \| **Package** \| Class \| **Use** \| **Tree** \| **Deprecated** \| **Index** \| **Help** | |  |
| **PREV PACKAGE**   **NEXT PACKAGE** | **FRAMES**    **NO FRAMES**     **All Classes** |  |


---
